# The gut–bone axis: mechanisms through which oleic acid regulates bone metabolism and its potential in preventing and treating osteoporosis

**DOI:** 10.3389/fnut.2026.1745125

**Published:** 2026-01-23

**Authors:** Minshun Zhu, Xianda Zhang, Jianhua Zhang, Jiaping Chen, Long Liang

**Affiliations:** 1 Department of Rehabilitation, Lu'an Hospital of Traditional Chinese Medicine, Lu'an, China; 2 Department of Orthopedics, Suzhou Hospital of Integrated Traditional Chinese and Western Medicine, Suzhou, China; 3 Department of Orthopedics, The First Affiliated Hospital of Anhui University of Chinese Medicine, Hefei, China

**Keywords:** bone metabolism, dietary sources, fatty acid metabolism, gut microbiota, gut–bone axis, oleic acid, osteoporosis

## Abstract

Osteoporosis is a prevalent metabolic bone disorder characterized by reduced bone mass and increased fracture risk, posing a growing global health burden. Dietary factors have emerged as important modulators of bone metabolism, among which the monounsaturated fatty acid (MUFA) oleic acid—abundant in olive oil and nuts—has attracted increasing attention. This review summarizes current evidence on the mechanisms through which oleic acid influences bone metabolism, with particular emphasis on the gut–bone axis as an integrative regulatory pathway. We describe how oleic acid modulates gut microbiota composition, reinforces intestinal barrier integrity, and influences the production of microbiota-derived metabolites, including short-chain fatty acids (SCFAs), bile acids, and indole derivatives. These metabolites act on bone remodeling through specific signaling pathways and receptors, such as free fatty acid receptors, bile acid receptors, and the aryl hydrocarbon receptor (AhR), thereby linking dietary fat intake to skeletal homeostasis. Preclinical and clinical evidence supporting the bone-protective effects of oleic acid–rich dietary patterns is critically evaluated, while existing gaps—particularly the limited availability of randomized controlled trials using purified oleic acid—are highlighted. Finally, challenges and future directions are discussed, including interindividual variability in gut microbiota, translational limitations, and the potential for personalized nutrition strategies. Collectively, current evidence suggests that oleic acid represents a promising dietary component for supporting bone health, with the gut–bone axis providing a mechanistic framework for future research and potential translational exploration.

## 1 Introduction

Osteoporosis is a systemic skeletal disorder characterized by low bone mineral density and deterioration of bone microarchitecture, resulting in increased bone fragility and fracture risk. Osteoporotic fractures are associated with substantial morbidity, mortality, and healthcare costs, particularly among aging populations and postmenopausal women. Epidemiological data indicate that osteoporosis represents a major global public health burden, with its prevalence continuing to rise worldwide (1). Despite advances in pharmacological therapies, long-term safety concerns, suboptimal adherence, and residual fracture risk highlight the need for complementary prevention and management strategies, including dietary and lifestyle interventions.

Growing evidence suggests that dietary components, particularly fatty acids, play an important role in regulating bone metabolism by influencing inflammation, energy homeostasis, and cellular differentiation pathways. Among these, oleic acid—the predominant monounsaturated fatty acid (MUFA) in olive oil and many nuts—has attracted increasing attention for its potential role in skeletal health. Experimental studies indicate that oleic acid can promote osteoblast differentiation and activity while inhibiting osteoclast formation, thereby favoring bone formation and preserving bone mass (2–4). In addition, the bioactivity of oleic acid may be influenced by its dietary source and processing methods; for example, cold-pressed olive oil retains higher levels of bioactive fatty acids compared with refined oils, which may partially account for differences in biological effects observed across studies (5, 6).

Beyond its direct effects on bone cells, oleic acid may exert broader metabolic actions relevant to skeletal homeostasis. Fatty acids function not only as energy substrates but also as signaling molecules that regulate inflammatory pathways and systemic metabolism. Increasing attention has been directed toward the gut microbiota as a key mediator of dietary fat–host interactions. The gut microbiota plays a central role in nutrient metabolism and immune regulation, and microbial fermentation products such as short-chain fatty acids (SCFAs) have been shown to support bone health by promoting osteoblast differentiation and suppressing osteoclastogenesis (2).

The concept of the gut–bone axis has therefore emerged as an integrative framework linking intestinal function, microbial metabolism, and skeletal remodeling. Dietary fatty acids, including oleic acid, can shape gut microbial composition and metabolic output, potentially influencing bone metabolism indirectly through microbiota-derived signaling molecules (2, 3). These interactions suggest that some of the skeletal benefits associated with oleic acid–rich dietary patterns may be mediated, at least in part, through modulation of the intestinal microenvironment and microbial metabolism.

In this review, we provide a comprehensive and structured overview of current knowledge regarding the role of oleic acid in bone metabolism, with particular emphasis on gut microbiota–mediated mechanisms. We first summarize the biological characteristics and metabolic pathways of oleic acid, followed by an analysis of its molecular effects on bone cells. We then examine how oleic acid interacts with the gut microbiota and microbial metabolites to influence bone remodeling through the gut–bone axis. Finally, we critically evaluate preclinical and clinical evidence, discuss translational challenges, and outline future research directions relevant to osteoporosis prevention and management.

## 2 Biological and molecular mechanisms of oleic acid in fat and bone metabolism

### 2.1 Structural characteristics and metabolic pathways of oleic acid

Oleic acid is a monounsaturated fatty acid (MUFA) consisting of an 18-carbon chain with a single cis double bond at the ninth carbon position (C18:1, n-9). This structural feature confers high membrane fluidity and biochemical stability, making oleic acid a major constituent of cellular membranes and a key regulator of lipid metabolism. In mammalian systems, oleic acid is synthesized endogenously through the fatty acid synthesis pathway. Acetyl-CoA serves as the initial substrate, which is converted to malonyl-CoA by acetyl-CoA carboxylase, followed by chain elongation catalyzed by fatty acid synthase. The conversion of stearic acid (C18:0) to oleic acid (C18:1) via stearoyl-CoA desaturase represents a critical regulatory step, introducing the defining cis double bond (7–9).

Beyond synthesis, oleic acid undergoes dynamic metabolic turnover. It can be esterified into triglycerides or phospholipids, contributing to lipid storage and membrane architecture, or degraded through mitochondrial β-oxidation to generate acetyl-CoA for energy production. Enzymes such as diacylglycerol acyltransferase regulate its incorporation into triglycerides, whereas phospholipid remodeling determines membrane composition and function. The balance between oleic acid and polyunsaturated fatty acids within membranes influences membrane integrity, receptor signaling, and cellular responsiveness to metabolic cues (10–12).

Disruption of oleic acid metabolism has been associated with metabolic disorders including obesity, insulin resistance, and cardiovascular disease, underscoring its importance in systemic metabolic regulation. Dietary intake, hormonal status, and energy availability jointly influence endogenous oleic acid synthesis and utilization, with stearoyl-CoA desaturase acting as a central metabolic node. Collectively, the structural properties and metabolic pathways of oleic acid provide the biochemical foundation for its diverse biological effects, including its emerging role in bone and fat metabolism (10, 13, 14).

### 2.2 Functions of oleic acid in adipocyte differentiation and systemic lipid metabolism

Oleic acid plays a significant role in adipocyte differentiation and lipid homeostasis as a representative MUFA. Experimental evidence indicates that oleic acid promotes the differentiation of preadipocytes into mature adipocytes through the regulation of key transcription factors, including CCAAT/enhancer-binding protein-α and peroxisome proliferator-activated receptor-γ. This process is accompanied by increased triglyceride synthesis and lipid droplet formation, reflecting enhanced lipid storage capacity within adipose tissue (15, 16).

In addition to promoting adipogenesis, oleic acid modulates lipid turnover by regulating enzymes involved in lipogenesis and lipolysis. It has been reported to suppress excessive fatty acid synthesis while facilitating adaptive lipid mobilization in response to energy demands, thereby contributing to metabolic flexibility. These effects are mediated, at least in part, through signaling pathways involving AMP-activated protein kinase and mammalian target of rapamycin, which coordinate cellular energy sensing and anabolic–catabolic balance (17, 18).

Oleic acid also influences bone marrow adipose tissue, a specialized fat depot increasingly recognized as an important regulator of skeletal health. Expansion of bone marrow adiposity is frequently associated with impaired bone formation and osteoporosis. Elevated levels of oleic acid, particularly in metabolic disorders such as obesity, may contribute to bone marrow adipose tissue remodeling, thereby indirectly influencing bone metabolism. This interaction highlights the close relationship between systemic lipid metabolism and skeletal homeostasis (19, 20).

At the systemic level, dietary oleic acid favorably modulates circulating lipid profiles by reducing low-density lipoprotein cholesterol and increasing high-density lipoprotein cholesterol, which contributes to improved metabolic health (21). Oleic acid also regulates the expression of genes involved in fatty acid uptake, transport, and storage, including those encoding peroxisome proliferator-activated receptors and fatty acid-binding proteins (22). Improved insulin sensitivity associated with oleic acid intake further links lipid metabolism to bone health, as insulin resistance and chronic inflammation are recognized contributors to bone loss (23).

Collectively, oleic acid functions as a key regulator of adipocyte differentiation, lipid metabolism, and metabolic syndrome–related processes. By influencing both adipose tissue biology and systemic metabolic health, oleic acid provides an important metabolic context for understanding its indirect effects on bone remodeling and osteoporosis risk (24–26).

### 2.3 Molecular mechanisms of oleic acid in regulating bone metabolism

Oleic acid has attracted increasing attention for its ability to modulate bone metabolism at the cellular and molecular levels. Bone marrow mesenchymal stem cells (BMSCs) represent a critical progenitor population that can differentiate into osteoblasts or adipocytes, and the balance between these lineages is central to skeletal health. Experimental studies suggest that oleic acid favors osteogenic differentiation of mesenchymal stem cells by activating signaling pathways involved in bone formation, including the Wnt/β-catenin pathway. Activation of this pathway enhances the expression of osteogenic markers such as Runx2 (runt-related transcription factor 2) and osteocalcin, thereby promoting osteoblast maturation and function (27).

In parallel, oleic acid suppresses excessive adipogenic differentiation within the bone marrow niche by modulating peroxisome proliferator-activated receptor-γ activity. Although peroxisome proliferator-activated receptor-γ is essential for lipid metabolism, its overactivation in mesenchymal stem cells shifts differentiation toward adipogenesis at the expense of osteogenesis. Oleic acid appears to attenuate this adipogenic bias, thereby preserving osteoblastogenic potential and limiting bone marrow adipose tissue expansion (16, 27).

Oleic acid also directly influences the activity of mature bone cells. Studies indicate that oleic acid promotes osteoblast proliferation and differentiation while indirectly inhibiting osteoclastogenesis by altering the osteoprotegerin (OPG)/receptor activator of nuclear factor-κB ligand ratio. Through the downregulation of receptor activator of nuclear factor-κB ligand expression and upregulation of OPG, oleic acid contributes to a microenvironment that favors bone formation over resorption (28–31).

Inflammatory signaling represents another key mechanism linking oleic acid to bone remodeling. Chronic low-grade inflammation enhances osteoclast differentiation and suppresses osteoblast activity, accelerating bone loss. Oleic acid exhibits anti-inflammatory effects by inhibiting nuclear factor-κB signaling and reducing the production of pro-inflammatory cytokines such as tumor necrosis factor-α and interleukin-6. These effects further support osteoblast function and restrain osteoclast activity, contributing to skeletal protection (32, 33).

**Figure 1:**
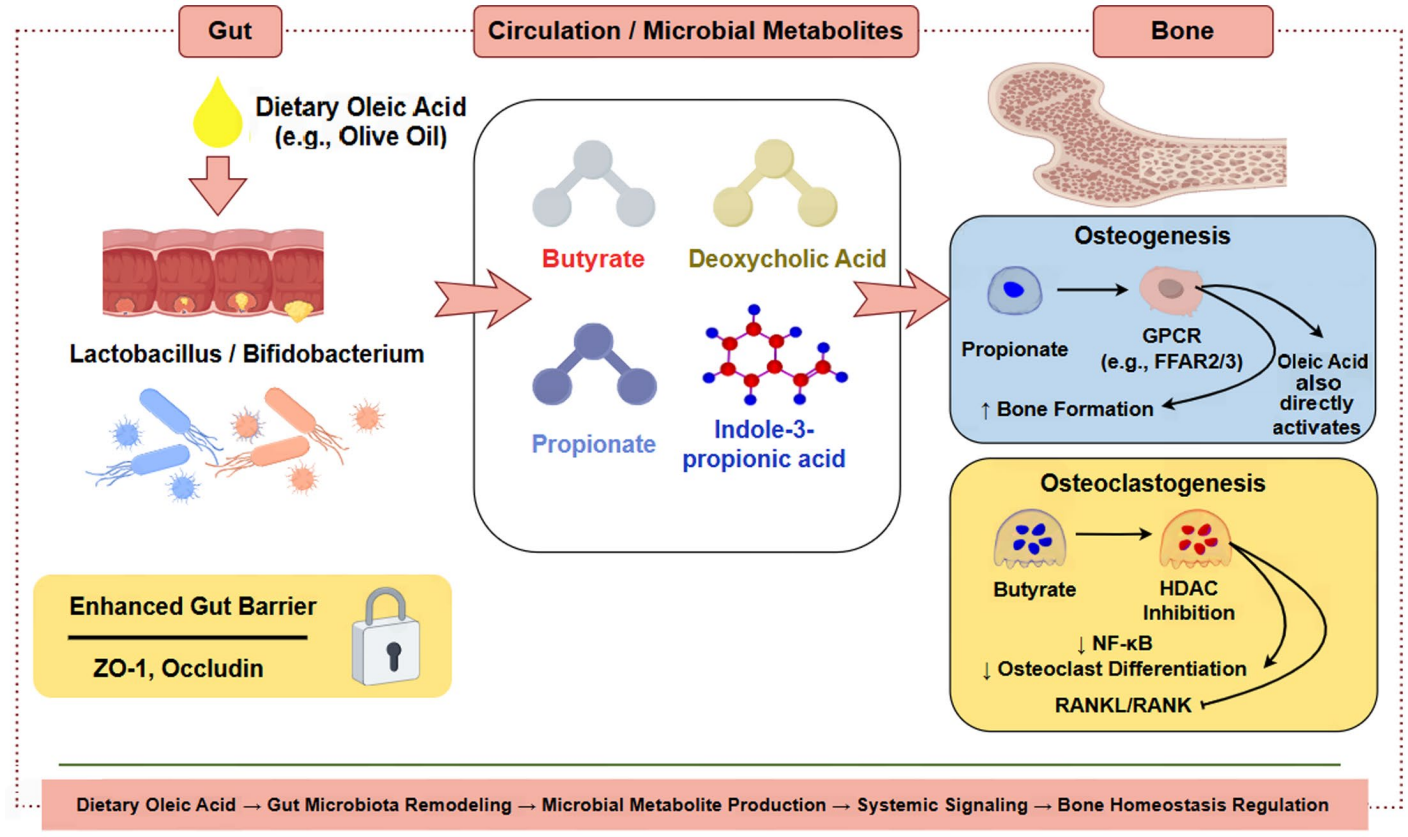
Molecular interplay in the oleic acid-modulated gut-bone axis. This schematic illustrates the signaling network through which dietary oleic acid regulates bone metabolism via the gut microbiota and their metabolites. In the gut lumen, oleic acid promotes beneficial bacteria and enhances barrier integrity. Microbial metabolites, including SCFAs, bile acids, and indoles, enter circulation. In bone tissue, these metabolites and oleic acid itself converge to promote osteoblastogenesis (e.g., via GPCRs and Wnt/β-catenin) while inhibiting osteoclastogenesis (e.g., via HDAC inhibition and AhR signaling), collectively maintaining bone homeostasis. SCFAs, short-chain fatty acids; GPCR, G protein-coupled receptor; HDAC, histone deacetylase; AhR, aryl hydrocarbon receptor. Figure drawn by Figdraw.com.

Collectively, oleic acid regulates bone metabolism through coordinated effects on mesenchymal stem cell fate, osteoblast and osteoclast activity, and inflammatory signaling pathways. These molecular mechanisms provide a mechanistic foundation for understanding how dietary oleic acid may contribute to the maintenance of bone homeostasis and the prevention of osteoporosis. An overview of oleic acid metabolism and the principal molecular pathways involved in bone cell regulation is summarized in Figure 1, Table 1.

**Table 1:** Molecular mechanisms of oleic acid in regulating bone metabolism.

Target process/cell	Mechanism of action	Key molecules/pathways involved	References
Osteoblast differentiation & activity	Promotes osteogenic differentiation of bone marrow mesenchymal stem cells and osteoblasts.	Wnt/β-catenin pathway; Runx2; Osteocalcin	(27)
Osteoclastogenesis	Indirectly inhibits osteoclast formation and activity.	Alters OPG/RANKL ratio; downregulates RANKL; upregulates OPG	(28–31)
Bone marrow adiposity & cell fate	Suppresses excessive adipogenic differentiation in bone marrow; favors osteogenic over adipogenic commitment.	Modulates PPARγ activity	(16, 27)
Inflammation	Exerts anti-inflammatory effects, creating a bone-forming microenvironment.	Inhibits NF-κB signaling; reduces TNF-α, IL-6 production	(32, 33)
Gut-bone axis signaling	Activates nutrient-sensing receptors; enhances intestinal barrier integrity.	Activates GPR40/GPR120 (FFAR1/FFAR4); upregulates tight junction proteins	(43–47)
Systemic metabolic & hormonal modulation	Modulates lipid metabolism and bone-anabolic hormone activity.	Improves insulin sensitivity; may influence IGF-1 signaling	(23, 42)

This table synthesizes the key molecular and cellular pathways through which oleic acid modulates bone remodeling, including its dual effects on enhancing osteoblastogenesis and suppressing osteoclastogenesis and marrow adipogenesis.

## 3 Gut microbiota-oleic acid-skeletal axis and its regulatory network

### 3.1 Metabolic and regulatory roles of gut microbiota in oleic acid handling

The gut microbiota plays a central role in regulating host lipid metabolism, including the absorption, transformation, and systemic effects of dietary oleic acid. Following ingestion, dietary oleic acid influences intestinal microbial communities primarily through indirect metabolic and inflammatory pathways, rather than through direct luminal interaction, as it is largely absorbed in the small intestine. Although oleic acid itself is not a primary substrate for colonic fermentation, changes in dietary fat quality can shape gut microbial composition and function by modulating host metabolism, bile acid profiles, and inflammatory signaling. Emerging evidence indicates that diets enriched in MUFAs, particularly oleic acid, are associated with alterations in gut microbial diversity and enrichment of bacterial taxa linked to metabolic homeostasis (34, 35).

Specific microbial communities, notably members of the Firmicutes and Bacteroidetes phyla, participate in lipid-related metabolic processes that influence host physiology. These bacteria contribute to the production of microbial metabolites, including SCFAs, which exert systemic effects despite being primarily derived from dietary fiber fermentation. Oleic acid–rich dietary patterns have been shown to increase the abundance of short-chain fatty acid–producing bacteria, thereby indirectly enhancing the availability of metabolites with known anti-inflammatory and metabolic regulatory properties (36, 37).

Beyond metabolite production, gut microbiota influence endocrine signaling pathways relevant to bone metabolism. Certain bacterial taxa regulate the secretion of gut-derived hormones such as glucagon-like peptide-1, which participates in glucose metabolism, appetite regulation, and systemic energy balance. These pathways highlight the role of the gut microbiota as an intermediary linking dietary lipid intake, including oleic acid, to host metabolic and skeletal regulation (38, 39).

### 3.2 Regulation of the intestinal microenvironment and microbial metabolism by oleic acid

Oleic acid plays an important role in maintaining intestinal homeostasis by modulating epithelial barrier integrity and inflammatory signaling within the gut microenvironment. Experimental studies indicate that oleic acid enhances the expression of tight junction proteins, thereby strengthening the intestinal barrier and limiting the translocation of luminal antigens and microbial products into the systemic circulation. Preservation of barrier integrity is particularly relevant in conditions characterized by chronic low-grade inflammation, which is a recognized contributor to osteoporosis pathogenesis (34, 40).

In parallel, oleic acid exerts anti-inflammatory effects within the intestinal milieu by suppressing pro-inflammatory cytokines such as tumor necrosis factor-α and interleukin-6. These actions help mitigate inflammation-associated dysbiosis and create a microbial environment conducive to metabolic balance. The interaction between oleic acid and the gut microbiota is bidirectional: oleic acid shapes microbial composition by promoting the growth of beneficial taxa, while microbial metabolism influences the systemic bioactivity of dietary oleic acid (3, 41).

A key downstream consequence of this interaction is the enhanced production of microbiota-derived metabolites, particularly SCFAs. Although oleic acid is not itself fermented into SCFAs, its ability to enrich short-chain fatty acid–producing bacteria indirectly amplifies metabolite availability. These metabolites exert systemic effects, including modulation of bone remodeling, thereby establishing a functional link between intestinal lipid handling and skeletal health (36).

**Figure 2:**
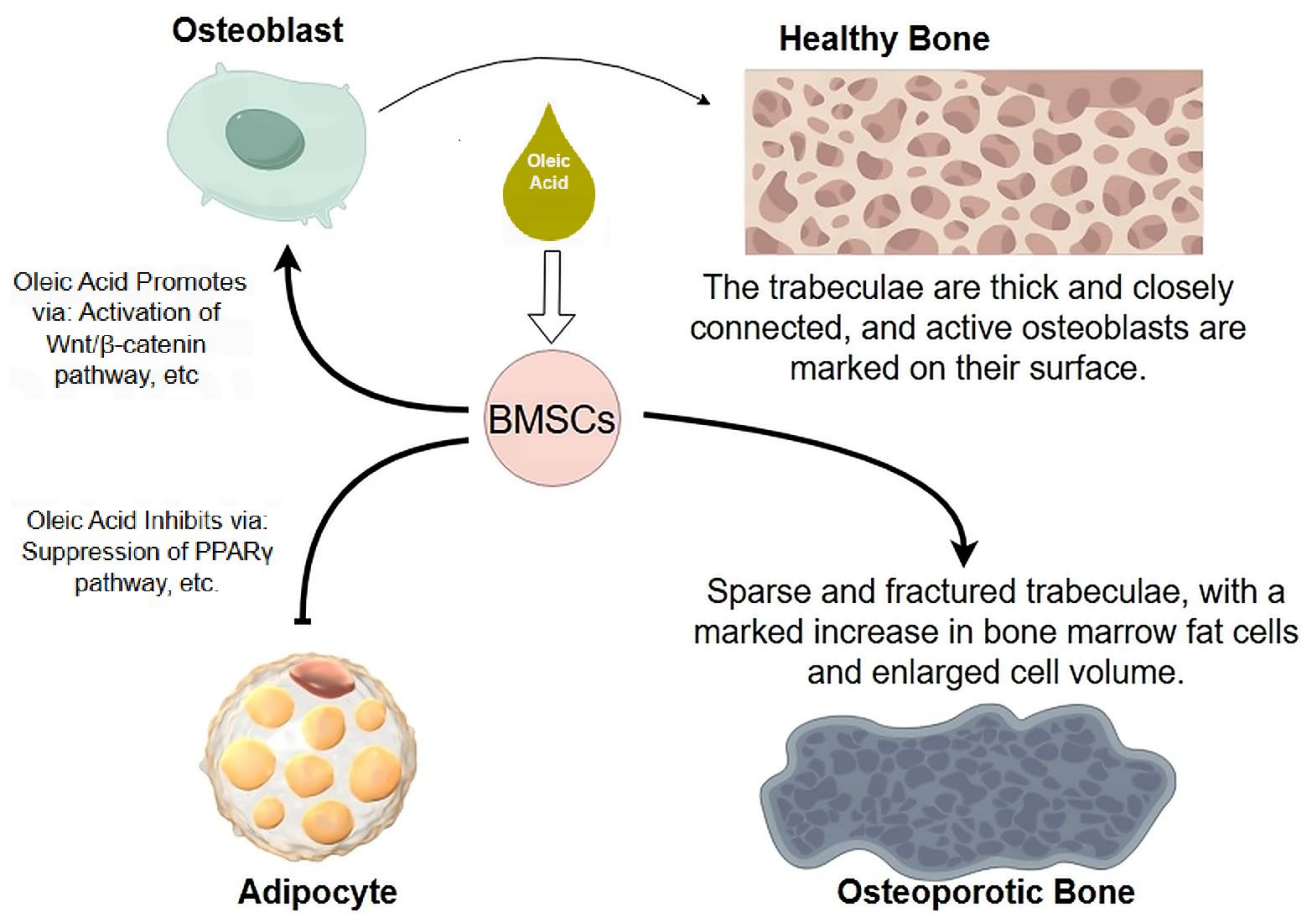
The dual regulation of BMSCs by oleic acid. This schematic illustrates how oleic acid guides the differentiation of BMSCs. At the differentiation crossroads, oleic acid promotes osteogenesis by activating the Wnt/β-catenin pathway and upregulating osteogenic factors (e.g., Runx2) (arrow), thereby enhancing bone formation. Conversely, oleic acid suppresses adipogenesis by inhibiting key regulators such as PPARγ (blunt arrow), reducing bone marrow fat deposition. By directing BMSCs toward osteoblast rather than adipocyte differentiation, oleic acid creates a bone marrow microenvironment conducive to bone mass accumulation. BMSCs, bone marrow mesenchymal stem cells; Runx2, Runt-related transcription factor 2; PPARγ, peroxisome proliferator-activated receptor gamma. Figure drawn by Figdraw.com.

Collectively, oleic acid contributes to the regulation of the intestinal microenvironment by reinforcing barrier function, suppressing inflammation, and shaping microbial metabolic output. These effects provide a mechanistic basis for understanding how dietary oleic acid may influence bone metabolism indirectly through gut-mediated pathways. The interactions between oleic acid, the gut microbiota, and the intestinal microenvironment within the gut–bone axis are schematically illustrated in Figure 2 and summarized in Table 2.

**Table 2:** Effects of oleic acid on the gut microbiota and intestinal environment.

Aspect of interaction	Observed effect of oleic acid	Downstream consequences/related mechanisms	References
Microbial composition	Enriches beneficial bacterial taxa; alters gut microbial ecology.	Increases microbial diversity; promotes a community conducive to metabolic homeostasis.	(34, 35)
Microbial metabolism & output	Indirectly promotes the growth of SCFA-producing bacteria.	Enhances the availability of SCFAs (e.g., butyrate, propionate).	(36, 37)
Intestinal barrier function	Strengthens the intestinal epithelial barrier.	Upregulates tight junction protein expression; reduces antigen translocation.	(34, 40)
Intestinal inflammation	Exerts local anti-inflammatory effects within the gut.	Suppresses pro-inflammatory cytokines (e.g., TNF-α, IL-6).	(3, 41)
Endocrine signaling	Influences gut hormone secretion via microbiota interaction.	Modulates secretion of hormones like glucagon-like peptide-1.	(38, 39)

This table summarizes the bidirectional interactions between oleic acid and the gut microbiome, highlighting how oleic acid shapes microbial composition and function, which in turn may influence bone health through metabolite production and anti-inflammatory effects.

### 3.3 Integration and translational potential of oleic acid signaling in the gut–liver–bone axis

The gut–liver–bone axis represents an integrated physiological network through which intestinal metabolism, hepatic lipid handling, and skeletal remodeling are coordinated. Within this framework, gut microbiota–derived metabolites serve as signaling intermediates that transmit dietary cues to distant organs. Experimental models demonstrate that alterations in gut microbial composition and fecal metabolomic profiles are accompanied by changes in bone density and microarchitecture, supporting the functional relevance of this axis in osteoporosis (27).

Oleic acid occupies a central position within the gut–liver–bone axis by acting as both a metabolic substrate and a signaling molecule. Following intestinal absorption, oleic acid undergoes hepatic metabolism and contributes to the generation of lipid-derived mediators that influence systemic inflammation and energy balance. In animal models, oleic acid–related metabolites, such as oleoyl serine, have been shown to promote bone formation and suppress bone resorption, thereby reversing bone loss and bone marrow adiposity (42).

At the molecular level, oleic acid engages multiple nutrient-sensing receptors along the gut–liver–bone continuum. In the intestine, oleic acid activates free fatty acid receptors, including GPR40 and GPR120, on enteroendocrine and immune cells, leading to the secretion of gut hormones and enhancement of barrier integrity. These signals indirectly influence bone metabolism by modulating systemic inflammation and nutrient availability (43, 44). Following systemic distribution, oleic acid and its derivatives can act directly on bone cells, where activation of GPR120 suppresses inflammatory signaling pathways such as nuclear factor-κB, thereby inhibiting osteoclastogenesis and favoring bone preservation (45–47).

Through coordinated engagement of membrane and nuclear receptors across multiple organs, oleic acid functions as an integrative molecular signal that translates dietary lipid intake into systemic effects on skeletal homeostasis. This multi-organ signaling network highlights the mechanistic plausibility of targeting dietary oleic acid within the gut–bone axis to support bone health.

### 3.4 Microbiota-derived metabolites as molecular mediators linking oleic acid to bone remodeling

Microbiota-derived metabolites constitute critical effector molecules linking dietary patterns to bone remodeling. Multi-omics studies demonstrate that diets enriched in oleic acid–rich oils reshape gut microbial ecology and fecal metabolomic profiles, including lipid-related metabolites that participate in systemic signaling (48). These findings support the concept that oleic acid–driven microbial remodeling precedes and facilitates downstream skeletal effects.

SCFAs represent the most extensively characterized class of microbial metabolites relevant to bone metabolism. Beyond their role as energy substrates for colonocytes, SCFAs act as signaling molecules by activating G-protein–coupled receptors and inhibiting histone deacetylases, thereby influencing immune regulation and gene expression. Experimental studies demonstrate that butyrate stimulates bone formation through regulatory T cell–dependent mechanisms involving WNT signaling, while simultaneously suppressing osteoclast differentiation and activity (49, 50).

In addition to SCFAs, bile acid signaling provides an important link between gut microbial metabolism and skeletal homeostasis. Microbial transformation of primary bile acids alters the circulating bile acid pool, enabling activation of bile acid receptors such as farnesoid X receptor and TGR5. Activation of these receptors has been shown to promote osteoblast differentiation and mineralization through pathways involving energy metabolism and inflammatory regulation (51, 52).

Tryptophan-derived indole metabolites further expand the spectrum of microbial signals influencing bone. These compounds activate the AhR, which regulates intestinal barrier function and systemic inflammation. Dysregulated AhR signaling has been associated with impaired bone formation and enhanced osteoclastogenesis, whereas modulation of this pathway improves skeletal outcomes in aging and inflammatory contexts (53–55).

**Figure 3:**
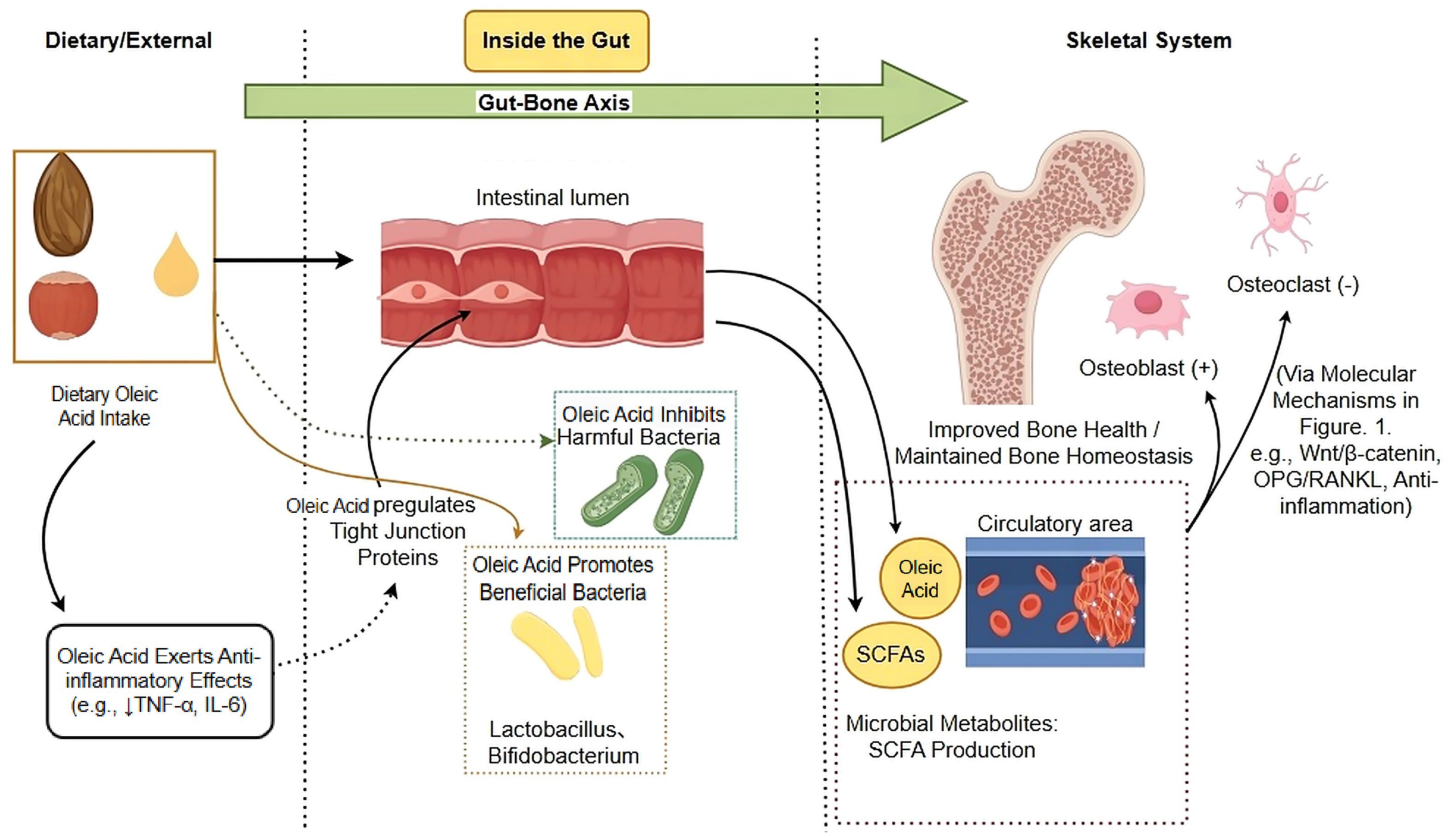
The gut–bone axis mediated by oleic acid and gut microbiota. This schematic illustrates how dietary oleic acid influences skeletal health, particularly in the context of osteoporosis, via modulation of the gut–bone axis. 1. Oleic acid actions on the gut: upon intake, oleic acid enhances intestinal barrier integrity (↑tight junction proteins) and exerts anti-inflammatory effects (↓TNF-α, IL-6) within the gut microenvironment. It also promotes a beneficial shift in gut microbiota composition (e.g., enrichment of *Lactobacillus*). 2. Microbial signal to bone: a key consequence of this microbiota remodeling is the increased production of SCFAs. Upon entering circulation, SCFAs act as systemic messengers that promote bone formation by stimulating osteoblast differentiation and inhibit bone resorption by suppressing osteoclastogenesis. 3. Systemic impact: this gut-derived signaling helps maintain bone mass and microarchitecture, thereby counteracting the pathophysiological processes of osteoporosis. The diagram highlights oleic acid as a key dietary regulator of this protective gut-bone crosstalk. SCFAs, short-chain fatty acids; TNF-α, tumor necrosis factor-alpha; IL-6, interleukin-6. Figure drawn by Figdraw.com.

Finally, intestinal barrier dysfunction facilitates translocation of microbial-associated molecular patterns, such as lipopolysaccharide, which activate inflammatory signaling cascades and promote osteoclastogenic cytokine production. By improving barrier integrity and limiting inflammatory translocation, oleic acid may indirectly suppress inflammation-driven bone resorption. Together, these metabolite- and immune-mediated pathways converge on key regulators of bone remodeling, integrating dietary oleic acid intake with gut microbiota activity and skeletal health. The integrated complex crosstalk among dietary oleic acid, gut microbiota, microbiota-derived metabolites, and bone remodeling pathways is depicted in Figure 3.

## 4 Experimental and clinical evidence for oleic acid in osteoporosis

### 4.1 Bone-protective effects of oleic acid in osteoporosis animal models

Preclinical studies using animal models of osteoporosis provide important mechanistic and proof-of-concept evidence supporting the bone-protective potential of oleic acid. In ovariectomized rodents, a widely used model of postmenopausal osteoporosis, interventions incorporating oleic acid have been shown to improve bone microarchitecture, bone strength, and trabecular connectivity. For example, delivery systems combining oleic acid with anabolic agents such as teriparatide have demonstrated enhanced therapeutic efficacy, suggesting that oleic acid may improve drug bioavailability and amplify anabolic signaling in bone tissue (56) (see Table 3).

**Table 3:** Preclinical and clinical evidence for the bone-protective effects of oleic acid.

Study model/type	Intervention/observation	Key findings related to bone health	References
Preclinical (animal models)	Oleic acid combined with teriparatide in nanoemulsion.	Improved bone microarchitecture, strength, and trabecular connectivity.	(56)
Preclinical (animal models)	Administration of oleic acid derivative (oleoyl serine).	Promoted bone formation, inhibited resorption, and reversed bone loss.	(42)
Preclinical (cellular & animal studies)	Oleic acid treatment.	Reduced bone marrow adiposity; promoted osteoblast activity and survival; inhibited osteoclastogenesis.	(2, 7, 31, 57, 58)
Observational (human studies)	High consumption of oleic acid-rich diets (e.g., Mediterranean diet).	Associated with greater bone mineral density.	(64)
Randomized controlled trials (RCTs)	Supplementation with extra virgin olive oil.	Improved bone-related parameters; associated with reduced fracture risk (PREDIMED trial).	(65, 66)
Dose & safety reference	Dietary intake from high-oleic oils.	~20 g/day of high-oleic oil (providing ~15 g oleic acid) is recognized as safe for cardiovascular health (FDA qualified claim).	(59)

This table consolidates empirical evidence from animal models and human studies, demonstrating the efficacy of oleic acid and its derivatives in improving bone metrics and their potential in osteoporosis management strategies.

In addition to its role as a formulation component, oleic acid itself appears to modulate skeletal remodeling *in vivo*. Animal studies indicate that oleic acid supplementation reduces inflammatory burden and supports osteoblast activity while restraining excessive osteoclast-mediated bone resorption. These effects are consistent with mechanistic findings described in cellular models, including modulation of lipid signaling and inflammatory pathways relevant to bone homeostasis (56). Collectively, animal data support the biological plausibility of oleic acid as a dietary or adjunctive factor in osteoporosis management, while also highlighting the need for cautious extrapolation to humans.

### 4.2 Regulation of bone marrow adiposity and bone cell dynamics by oleic acid

Bone marrow adiposity is increasingly recognized as a critical determinant of skeletal health, with excessive marrow fat accumulation being negatively associated with bone mineral density and bone strength. Experimental evidence suggests that oleic acid influences the bone marrow microenvironment by modulating the balance between adipogenic and osteogenic differentiation. Studies in animal and cellular models indicate that oleic acid reduces bone marrow adipocyte accumulation while promoting osteoblast proliferation and differentiation, thereby favoring bone formation (2, 7).

At the cellular level, oleic acid supports osteoblast survival and function by reducing apoptosis and sustaining anabolic activity. Importantly, oleic acid does not induce transdifferentiation of osteoblasts into osteoclasts. Instead, its effects on osteoclastogenesis are indirect and context dependent, mediated through alterations in inflammatory signaling, lipid metabolism, and the OPG/receptor activator of nuclear factor-κB ligand axis (31, 57). Several studies report inhibitory effects of oleic acid on osteoclast differentiation and bone resorption, although these effects may vary depending on experimental conditions and the local inflammatory milieu (58).

**Figure 4:**
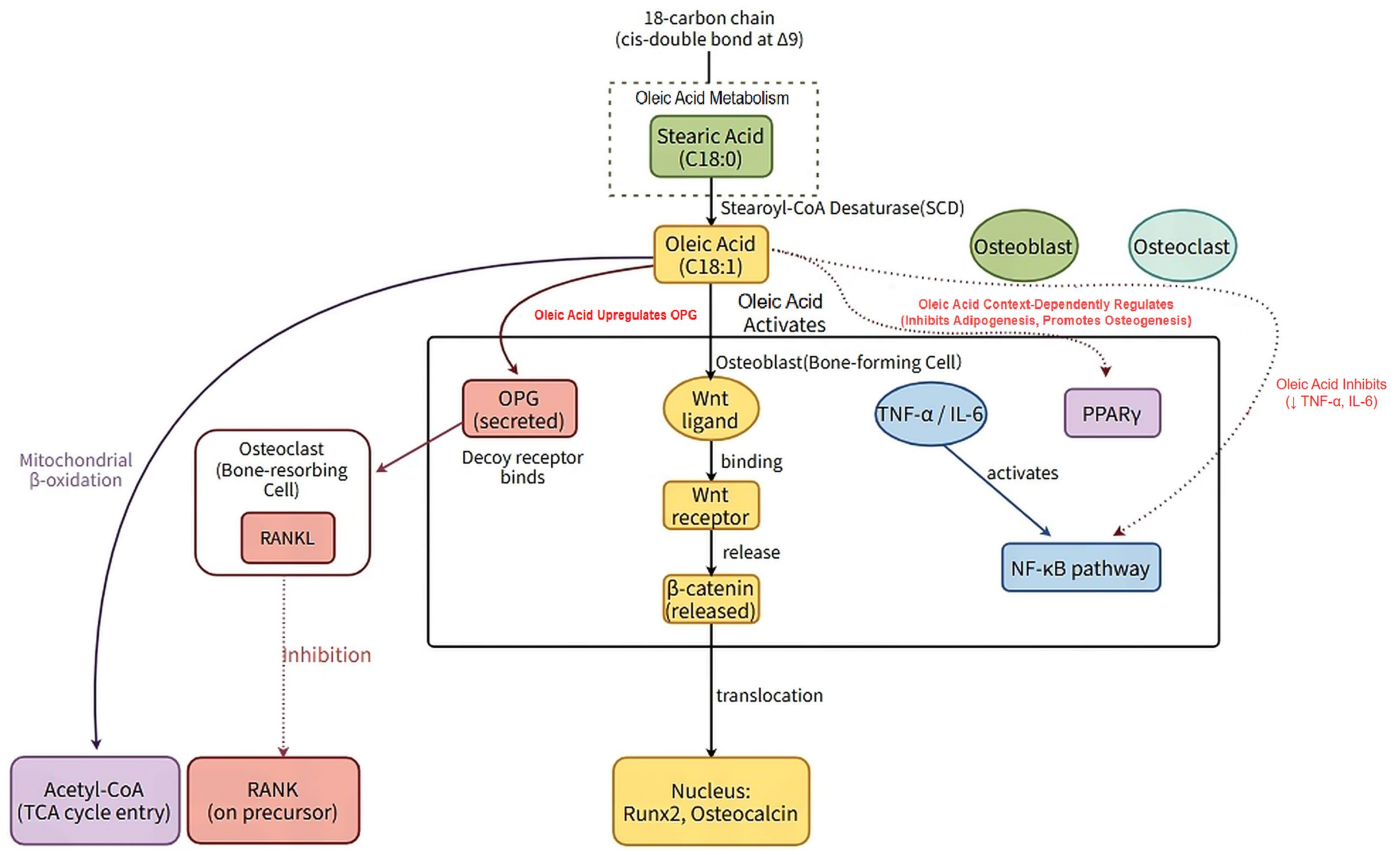
Oleic acid metabolism and key molecular pathways in bone cells. Oleic acid exerts dual regulatory effects on bone homeostasis. The left panel (purple, red, and yellow themes) illustrates how oleic acid promotes osteoblast activity through the following pathways: (1) activation of the Wnt/β-catenin signaling pathway, leading to nuclear accumulation of β-catenin and upregulation of osteogenic genes (e.g., Runx2, osteocalcin); (2) increasing OPG expression; (3) exerting an anti-apoptotic effect on osteoblasts. The right panel (green, blue, and purple themes) illustrates oleic acid’s inhibition of osteoclast generation and function through the following mechanisms: (1) downregulating RANKL expression in osteoblasts, thereby suppressing the RANKL/RANK signaling pathway essential for osteoclast differentiation; (2) inhibits the NF-κB inflammatory pathway, leading to reduced secretion of pro-inflammatory cytokines (e.g., TNF-α, IL-6). This schematic illustrates the central role of oleic acid in shifting the balance of bone remodeling toward bone formation. OPG, osteoprotegerin; Runx2, Runt-related transcription factor 2; RANKL, Receptor Activator of NF-κB Ligand; RANK, Receptor activator of NF-κB; NF-κB, Nuclear Factor Kappa-B. Figure drawn by Figdraw.com.

Together, these findings underscore the role of oleic acid as a regulator of bone cell dynamics and marrow adiposity rather than a direct determinant of osteoclast lineage commitment. Modulation of the bone marrow niche by dietary fatty acids represents a plausible mechanism through which oleic acid may contribute to skeletal preservation in aging and hormone-deficient states. Figure 4 summarizes oleic acid’s key role in the bone marrow microenvironment: guiding stem cells toward osteoblast differentiation rather than adipocyte differentiation.

### 4.3 Dose–response relationship and safety considerations of oleic acid

#### 4.3.1 Intake levels and reference values

Oleic acid is widely consumed as a component of dietary fats, particularly in olive oil–rich dietary patterns. However, clinical guidance regarding isolated oleic acid supplementation remains limited. Regulatory authorities provide indirect reference values based on cardiovascular outcomes. The U.S. Food and Drug Administration has issued a qualified health claim indicating that daily consumption of approximately 1.5 tablespoons (≈20 g) of oils containing a high proportion of oleic acid may reduce the risk of coronary heart disease when replacing saturated fats (59). While these recommendations are not osteoporosis-specific, they provide a pragmatic benchmark for considering safety in nutritional contexts.

#### 4.3.2 Adverse effects and toxicity thresholds

At habitual dietary intake levels, oleic acid is generally well tolerated. Most safety data derive from dietary exposures rather than purified formulations. In contrast, concentrated or pharmaceutical applications of oleic acid—such as lipid-based drug delivery systems—require careful dose optimization to minimize adverse effects, including gastrointestinal discomfort or alterations in lipid metabolism (60, 61). To date, no definitive maximum tolerated dose or no-observed-adverse-effect level has been established for oleic acid supplementation in humans, underscoring the need for systematic dose-escalation studies.

#### 4.3.3 Interactions with calcium, vitamin D, and osteoporosis therapies

Calcium and vitamin D remain foundational components of osteoporosis management, particularly in postmenopausal women (62). Dietary fat composition can influence intestinal calcium absorption, although the specific effects of MUFAs such as oleic acid on calcium bioavailability remain incompletely characterized (63). Moreover, there is currently no direct clinical evidence indicating that oleic acid interferes with or enhances the efficacy of established osteoporosis medications, including bisphosphonates, denosumab, or teriparatide. Nevertheless, given oleic acid’s effects on lipid signaling and inflammation, potential nutrient–drug interactions warrant consideration in future clinical trials.

### 4.4 Epidemiological and clinical translational evidence

#### 4.4.1 Observational evidence from oleic acid–rich dietary patterns

Epidemiological studies examining dietary fat quality suggest that dietary patterns rich in MUFAs, particularly olive oil–based diets, are associated with favorable bone health outcomes. For example, a cross-sectional study in Spanish women reported that higher olive oil consumption (>18.32 g/day) was associated with greater total, trabecular, and cortical bone density compared with lower intake levels (64).

Importantly, these findings reflect complex dietary patterns rather than isolated oleic acid exposure. Given the observational nature of such studies and the presence of multiple bioactive components in olive oil, conclusions regarding the independent effects of oleic acid should be interpreted with caution. These limitations highlight the need to distinguish overall dietary pattern effects from the specific biological actions of individual fatty acids.

#### 4.4.2 Randomized controlled trials using olive oil–based interventions

Randomized controlled trials provide more robust evidence linking oleic acid–rich dietary exposures to skeletal outcomes. In a randomized intervention study involving severely obese adults, supplementation with extra virgin olive oil for 12 weeks resulted in measurable changes in bone-related parameters, including markers of mineral metabolism (65). In addition, long-term data from the PREDIMED trial demonstrated that higher extra virgin olive oil consumption was associated with a significantly reduced risk of osteoporotic fractures, supporting a protective association between olive oil–rich dietary patterns and skeletal health (66).

While these trials do not isolate oleic acid from other olive oil constituents, they provide clinically relevant evidence supporting the translational potential of oleic acid–rich diets in bone health maintenance.

#### 4.4.3 Evidence gaps and future randomized trial design

Despite supportive observational and dietary intervention data, direct evidence evaluating purified oleic acid supplementation in patients with osteoporosis is lacking. Existing studies predominantly assess olive oil matrices, in which biological effects may reflect combined actions of oleic acid and minor components such as polyphenols (65). Future randomized controlled trials should therefore aim to disentangle these effects by comparing purified oleic acid with oleic acid–rich oils, employing dose-ranging designs, and incorporating both skeletal endpoints and gut microbiota–related biomarkers.

### 4.5 Biomarker potential and risk stratification in personalized medicine

Oleic acid and its metabolites have emerged as potential biomarkers linking lipid metabolism to skeletal health. Advances in metabolomics and lipid profiling enable the quantification of circulating fatty acids and their derivatives, offering opportunities for improved osteoporosis risk stratification. Preclinical studies indicate that oleic acid–derived mediators, such as oleoyl serine, exert bone-protective effects and may serve as candidate biomarkers of anabolic bone activity (42).

Integration of lipidomic, genetic, and microbiome data may further enhance personalized approaches to osteoporosis prevention and management. By identifying individuals with altered fatty acid metabolism or heightened inflammatory susceptibility, oleic acid–centered dietary interventions could be tailored to maximize skeletal benefit while minimizing risk.

## 5 Future directions and translational challenges

Despite substantial progress in elucidating the molecular and cellular mechanisms linking dietary lipids to skeletal health, several critical challenges remain before oleic acid–centered strategies can be translated into routine clinical practice for osteoporosis prevention and management. A major limitation of current evidence is the predominance of *in vitro* and animal studies, which, although mechanistically informative, cannot fully recapitulate the complexity of human physiology. In particular, interindividual variability in metabolism, hormonal status, and gut microbiota composition introduces substantial heterogeneity in responses to dietary oleic acid, complicating direct extrapolation from preclinical models to human populations.

Future research should therefore prioritize well-designed human studies that integrate metabolic, skeletal, and microbiome-related endpoints. Randomized controlled trials specifically evaluating purified oleic acid—rather than olive oil matrices alone—will be essential to disentangle the independent effects of oleic acid from those of coexisting bioactive components, such as polyphenols. Such trials should incorporate dose-ranging designs, standardized dietary controls, and comprehensive safety monitoring, while assessing both classical skeletal outcomes (e.g., bone mineral density and fracture incidence) and mechanistically informative biomarkers, including bone turnover markers, inflammatory mediators, and gut-derived metabolites.

Advances in multi-omics technologies provide an opportunity to deepen mechanistic insight and refine translational relevance. Integrated analyses combining lipidomics, metabolomics, transcriptomics, and microbiome profiling may help identify molecular signatures associated with favorable skeletal responses to oleic acid intake. These approaches could also facilitate the identification of responder subgroups, thereby supporting the development of precision nutrition strategies tailored to individual metabolic and microbial profiles.

Another important translational challenge lies in regulatory and practical considerations. Oleic acid is widely consumed as part of habitual diets and is generally regarded as safe; however, its long-term effects when administered in concentrated or supplemental forms remain insufficiently characterized. Establishing standardized formulations, defining optimal intake ranges, and clarifying potential interactions with osteoporosis pharmacotherapies will be necessary before oleic acid–based interventions can be recommended in clinical guidelines.

Collectively, addressing these challenges will require interdisciplinary collaboration spanning nutrition science, bone biology, microbiome research, and clinical medicine. By integrating mechanistic understanding with rigorous clinical validation, future studies may enable the rational incorporation of oleic acid–centered dietary strategies into comprehensive approaches for maintaining skeletal health across the lifespan.

## 6 Conclusion

Oleic acid, a predominant MUFA in olive oil and related dietary patterns, has emerged as a biologically plausible modulator of bone metabolism. Accumulating evidence indicates that oleic acid influences skeletal homeostasis through multiple, interconnected mechanisms, including regulation of bone cell differentiation and activity, modulation of inflammatory signaling, and indirect effects mediated by the gut microbiota and its metabolites. These pathways converge on key regulators of bone remodeling, providing a coherent mechanistic framework linking dietary fat quality to skeletal health.

Experimental and observational studies support an association between oleic acid–rich dietary patterns and favorable bone outcomes, while randomized trials of olive oil–based interventions offer preliminary translational evidence. However, direct clinical data evaluating purified oleic acid supplementation in osteoporosis remain limited, underscoring the need for cautious interpretation and further investigation. Importantly, current findings suggest that the skeletal effects attributed to oleic acid likely reflect both its intrinsic biological actions and its integration within broader dietary and metabolic contexts.

In summary, oleic acid represents a promising dietary component for supporting bone health, particularly when considered within the gut–bone axis framework. Future research integrating multi-omics approaches, microbiome science, and rigorously designed clinical trials will be essential to clarify its therapeutic potential and define its role within evidence-based personalized strategies for osteoporosis prevention and management.
